# Correlation study of tumor-infiltrating lymphocytes combined with residual cancer burden and prognosis in breast cancer patients receiving neoadjuvant chemotherapy

**DOI:** 10.3389/fonc.2025.1538326

**Published:** 2025-09-09

**Authors:** Zexin Hou, Xueyuan An, Guangmin Meng, Hongmei Zhao, Shanghua Liao, Xiaomin Long, Lingjun Zou, Wen Wu, Li Feng, Guanghui Liao

**Affiliations:** ^1^ Department of Oncology, The Second Affiliated Hospital, Guizhou Medical University, Kaili, China; ^2^ Department of Pathology, Shengli Oilfield Central Hospital, Dong ying, China; ^3^ Department of Pathology, The Second Affiliated Hospital, Guizhou Medical University, Kaili, China; ^4^ Department of Oncology, Yibin First People's Hospital, Yibin, China; ^5^ Xinzhou Town Center Health Center Internal Medicine, Huangping, China

**Keywords:** breast cancer, tumor-infiltrating lymphocytes, residual cancer burden, disease-free survival, neoadjuvant chemotherapy

## Abstract

**Purpose:**

This study investigates the feasibility of utilizing a combination of tumor-infiltrating lymphocytes (TILs) and residual cancer burden (RCB) to predict the prognosis of breast cancer (BC) individuals post-neoadjuvant chemotherapy (NAC).

**Methods:**

Patients with BC who underwent surgery following NAC were recruited from three medical centers for this research. RCB and TIL levels were determined using established guidelines, and the integration of RCB and TIL assessments was termed “RCB-TILs”. The relationship between RCB-TILs and patients’ clinicopathological variables was analyzed, alongside the link between RCB-TILs and disease-free survival (DFS).

**Results:**

The study comprised 242 BC patients who underwent NAC, among whom 98 were identified as RCB-TILs (+), while 144 were classified as RCB-TILs (-). Multivariate analysis demonstrated that RCB-TILs (+) served as an independent factor impacting recurrence following NAC across all BC patients (hazard ratio [HR] = 0.225, 95% confidence interval [CI]: 0.099 – 0.508, P < 0.001), including hormone receptor-positive patients (HR = 0.213, 95%CI: 0.067 – 0.682, P = 0.009), HER2-positive patients (HR = 0.216, 95%CI: 0.048 – 0.968, P = 0.045), and those with triple-negative BC (HR = 0.220, 95%CI: 0.049 – 0.989, P = 0.048).

**Conclusions:**

RCB-TILs (+) are correlated with extended DFS in BC patients who have undergone surgery post-NAC. In these individuals, RCB-TILs may provide a more sensitive predictor of DFS than RCB or TILs individually.

## 1 Introduction

Breast cancer (BC) represents a prevalent malignant tumor among women, with both incidence and mortality rates ranking prominently in the World Cancer Spectrum. The majority of patients receive a diagnosis of lymph node metastasis, posing a significant threat to women’s health (1). Neoadjuvant chemotherapy (NAC) serves as a vital treatment modality for BC, capable of modifying the tumor microenvironment and impacting cancer cell viability (2). NAC is intended to downstage tumors, enhance surgical options, mitigate the risk of postoperative recurrence, and furnish data on drug sensitivity to inform subsequent treatment strategies (3). Nonetheless, due to the aggressive nature of tumor cells and their propensity for recurrence and metastasis, some patients experience unfavorable prognoses (4). Thus, monitoring the prognosis of BC patients undergoing NAC treatment holds considerable scientific and clinical value.

Prior research has indicated (5) that the residual cancer burden (RCB) index, which incorporates various elements such as the proportion of residual tumor cells and lymph node metastasis, serves as a tool to evaluate residual disease in BC patients post-surgery. Yau C et al. (6), through meta-analysis, have demonstrated that RCB is a significant determinant impacting the prognosis of BC patients. Nevertheless, the assessment of RCB focuses solely on residual disease status, neglecting the host immune response, potentially introducing bias into the accuracy of prognostic analysis. The adaptive immune response mediated by tumor-infiltrating lymphocytes (TILs) is crucial for effective and sustained anti-tumor activity. Within the tumor microenvironment, TILs are believed to play significant roles in immune response and regulation of tumor immune mechanisms (7). TILs correlate with treatment response and survival outcomes in various solid tumors and can predict disease-free survival (DFS) in cancer patients (8–10). Thus, the integration of RCB and TILs might provide more valuable prognostic insights. In this investigation, a novel “RCB-TILs” metric was established by integrating RCB and TILs, and its feasibility in predicting the prognosis of BC patients following NAC was assessed.

## 2 Materials and methods

### 2.1 Patients

A cohort of 242 individuals diagnosed with BC who received NAC prior to surgical procedures between January 2015 and December 2019 were incorporated in this investigation. These subjects were treated at three medical centers: the Second Affiliated Hospital of Guizhou Medical University, Shengli Oilfield Central Hospital, and the First People’s Hospital of Yibin. Diagnoses of stage II-III BC were made for all participants on the basis of the 8th edition of the American Joint Committee on Cancer TNM staging manual (11). Comprehensive clinical and pathological data were collected, encompassing age, histological characteristics, lymph node metastasis, and molecular subtypes. Prior to NAC, invasive BC was confirmed in the subjects through pathological biopsy. Surgical treatment was conducted following standard NAC regimens, and postoperative adjuvant therapy was tailored to each BC subtype. This study complied with the Declaration of Helsinki, with ethical approval obtained from the ethics committees of the three medical centers (approval No. 2023-Ethical Review-229). Informed consent was also secured from all participants involved in the study.

### 2.2 Molecular subtypes of BC

BC molecular subtypes were classified utilizing the immunohistochemical expression profiles of estrogen receptor (ER), progesterone receptor (PR), human epidermal growth factor receptor 2 (HER2), and Ki67 (12). These subtypes are defined as follows: Luminal A, characterized by positivity for ER and PR with PR positivity of ≥ 20%, negative HER2, and Ki67 < 14%; Luminal B, which includes ER-positive, HER2-negative cases with any PR and Ki67 expression or those with ER positivity, PR negativity or PR < 20%, HER2 negativity, and Ki67 ≥ 14%; HER2-positive breast cancer (HER2BC), defined by HER2 positivity and ER/PR negativity; and triple-negative breast cancer (TNBC), marked by the absence of ER, PR, and HER2. For this study, Luminal A and Luminal B subtypes were grouped under hormone receptor-positive breast cancer (HRBC).

### 2.3 Histopathological evaluation of TILs

The histopathological evaluation of TILs was carried out in accordance with the International Immuno-Oncology Biomarker Working Group report (7) on sections of core needle biopsy specimens that were stained with hematoxylin and eosin (H&E) and obtained at diagnosis. Two pathologists independently conducted the assessment. Following the established criteria, the extent of mononuclear inflammatory cell infiltration surrounding the invasive tumor cell nests relative to the stromal area was categorized as ≥ 50%, 10% – 50%, or ≤ 10%. Cases exhibiting ≤ 10% infiltration were deemed TILs-negative, whereas those with greater infiltration were classified as TILs-positive.

### 2.4 Histopathological evaluation of RCB

As per the guidelines of the MD Anderson Cancer Center (13), the RCB is computed using the formula: RCB = 1.4 (proportion of invasive cancer × primary tumor diameter) 0.17 + [4 (1−0.75 number of positive lymph nodes) × largest metastasis diameter]0.17. The outcomes are divided into three distinct categories: minimal residual disease (RCB-I), moderate residual disease (RCB-II), and extensive residual disease (RCB-III). Given the more favorable prognosis linked with RCB-I in comparison to RCB-II and RCB-III, RCB-I is regarded as RCB-positive, whereas the latter are categorized as RCB-negative.

### 2.5 RCB-TILs assessment

RCB and TILs were combined as “RCB-TILs”. Cases exhibiting both positive RCB and TILs are classified as RCB-TILs positive [RCB-TILs (+)], whereas cases in which either RCB or TILs are negative are deemed RCB-TILs negative [RCB-TILs (-)].

### 2.6 Response assessment

The main outcome measure of the investigation was DFS, defined as the interval from surgical intervention to the recurrence of the disease (whether local or distant), death due to any cause, or the final follow-up.

### 2.7 Statistical analysis

The statistical analyses were executed utilizing SPSS 22.0 (IBM Corp., Armonk, USA). The relationships between various RCB-TIL levels and clinicopathological parameters were evaluated via the chi-square test. Survival outcomes were assessed employing Kaplan-Meier curves and contrasted utilizing the log-rank test. For the Cox regression analysis, an initial univariate analysis of the variables was conducted, followed by a multivariate analysis. The assessment metrics included the hazard ratio (HR) and the 95% confidence interval (CI). Statistical significance was established utilizing a threshold of P < 0.05.

## 3 Results

### 3.1 Relationship between RCB-TILs and clinicopathological characteristics of NAC BC patients

**Figure 1:**
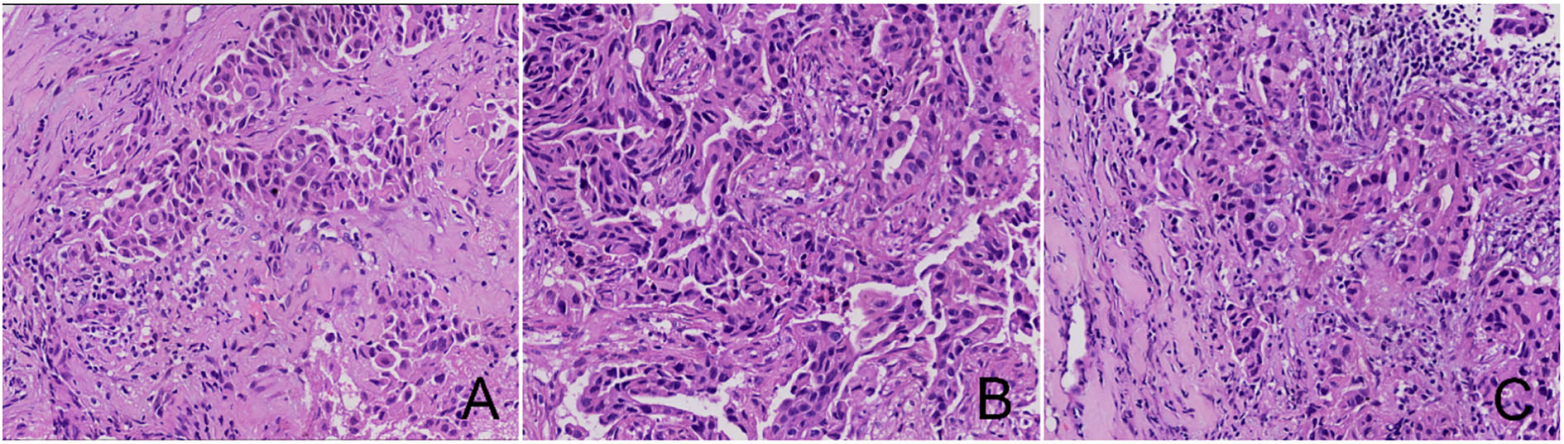
HE staining of TILs in breast cancer **(A)** TILs < 10%; **(B)** TILs 10%~50%; **(C)** TILs ≥50%.

**Table 1** displays the baseline characteristics of 242 participants (**Figure 1** illustrates the HE staining of TILs). Among these participants, 98 (40.5%) were identified as RCB-TILs (+), whereas 144 (59.5%) were classified as RCB-TILs (-). RCB-TILs (+) was correlated with reduced vascular invasion (P = 0.011), a decreased number of lymph node metastases (P = 0.008), a smaller proportion of HER2BC (P = 0.024), and an elevated pathological complete response (PCR) rate (P = 0.014) compared to RCB-TILs (-). Moreover, analyses were conducted separately for each subtype. Within the HRBC subgroup, individuals with RCB-TILs (+) showed a diminished risk of lymph node metastasis (P = 0.019) and an increased PCR rate (P = 0.002), while in the HER2BC subgroup, RCB-TILs (+) was linked to reduced vascular invasion (P = 0.021) (**Table 2**).

**Table 1:** Relationship between RCB-TILs and clinicopathological characteristics of NAC BC patients.

Characteristics	Number of patients	RCB-TILs (-)	RCB-TILs (+)	P value
All patients	242	144 (59.5%)	98 (40.5%)	
Age (years)				0.894
<60	144	85 (35.1%)	59 (24.4%)	
≥60	98	59 (24.4%)	39 (16.1%)	
Post-menopausal state				0.896
Yes	117	69 (28.5%)	48 (19.8%)	
No	125	75 (31.0%)	50 (20.7%)	
Tumor size				0.249
<2cm	69	37 (15.3%)	32 (13.2%)	
≥2cm	173	107 (44.2%)	66 (27.3%)	
Vascular invasion				0.011
Yes	37	29 (12.0%)	8 (3.3%)	
No	205	115 (47.5%)	90 (37.2%)	
Histological grade				0.713
I-II	207	122 (50.4%)	85 (35.1%)	
III	35	22 (9.1%)	13 (5.4%)	
Positive lymph node				0.008
Yes	99	69 (28.5%)	30 (12.4%)	
No	143	75 (31.0%)	68 (28.1%)	
Ki-67				0.316
<14%	71	46 (19.0%)	25 (10.3%)	
≥14%	171	98 (40.5%)	73 (30.2%)	
Pathological response				0.014
PCR	48	21 (8.7%)	27 (11.2%)	
non-PCR	194	123 (50.8%)	71 (29.3%)	
Molecular subtype				0.067
HRBC	124	81 (33.5%)	43 (17.8%)	
non-HRBC	118	63 (26.0%)	55 (22.7%)	
Molecular subtype				0.024
HER2BC	62	29 (12.0%)	33 (13.6%)	
non-HER2BC	180	115 (47.5%)	65 (26.9%)	
Molecular subtype				0.878
TNBC	56	34 (14.0%)	22 (9.1%)	
non-TNBC	186	110 (45.5%)	76 (31.4%)	

RCB-TILs (Tumor infiltrating lymphocytes - residual tumor load), PCR (Pathological complete response), HRBC (Hormone receptor-positive breast cancer), HER2BC (Human epidermal growth factor receptor 2-enriched breast cancer), TNBC (Triple-negative breast cancer).

**Table 2:** Relationship between RCB-TILs and clinicopathological characteristics of NAC BC patients with different subtypes.

Characteristics	HRBC (n=124)		P value	HER2BC (n=62)		P value	TNBC (n=56)		P value
	RCB-TILs (-)	RCB-TILs (+)		RCB-TILs (-)	RCB-TILs (+)		RCB-TILs (-)	RCB-TILs (+)	
Age (years)			0.691			0.611			0.786
<60	53 (42.7%)	30 (24.2%)		13 (21.0%)	18 (29.0%)		19 (34.0%)	11 (19.6%)	
≥60	28 (22.6%)	13 (10.5%)		16 (25.8%)	15 (24.2%)		15 (26.8%)	11 (19.6%)	
Post-menopausal state			1.000			1.000			0.577
Yes	30 (24.2%)	16 (12.9%)		17 (27.4%)	20 (32.2%)		22 (39.3%)	12 (21.4%)	
No	51 (41.1%)	27 (21.8%)		12 (19.4%)	13 (21.0%)		12 (21.4%)	10 (17.9%)	
Tumor size			0.219			1.000			0.401
<2cm	21 (16.9%)	16 (12.9%)		6 (9.7%)	7 (11.3%)		10 (17.9%)	9 (16.1%)	
≥2cm	60 (48.4%)	27 (21.8%)		23 (37.1%)	26 (41.9%)		24 (42.8%)	13 (23.2%)	
Vascular invasion			0.132			0.021			1.000
Yes	17 (13.7%)	4 (3.2%)		7 (11.3%)	1 (1.6%)		5 (8.9%)	3 (5.4%)	
No	64 (51.6%)	39 (31.5%)		22 (35.5%)	32 (51.6%)		29 (51.8%)	19 (33.9%)	
Histological grade			1.000			0.283			0.780
I-II	76 (61.3%)	41 (33.1%)		23 (37.1%)	30 (48.4%)		23 (41.1%)	14 (25.0%)	
III	5 (4.0%)	2 (1.6%)		6 (9.7%)	3 (4.8%)		11 (19.6%)	8 (14.3%)	
Positive lymph node			0.024			0.430			0.535
Yes	48 (38.7%)	16 (12.9%)		12 (19.4%)	10 (16.1%)		9 (16.1%)	4 (7.2%)	
No	33 (26.6%)	27 (21.8%)		17 (27.4%)	23 (37.1%)		25 (44.6%)	18 (32.1%)	
Ki-67			0.088			0.598			0.383
<14%	25 (20.2%)	7 (5.6%)		9 (14.4%)	13 (21.0%)		12 (21.4%)	5 (8.9%)	
≥14%	56 (45.2%)	36 (29.0%)		20 (32.3%)	20 (32.3%)		22 (39.3%)	17 (30.4%)	
Pathological response			0.004			0.244			0.329
PCR	7 (5.6%)	13 (10.5%)		5 (8.1%)	11 (17.7%)		9 (16.1%)	3 (5.4%)	
non-PCR	74 (59.7%)	30 (24.2%)		24 (38.7%)	22 (35.5%)		25 (44.6%)	19 (33.9%)	

### 3.2 Prognostic analysis of NAC BC patients based on RCB-TILs

**Figure 2:**
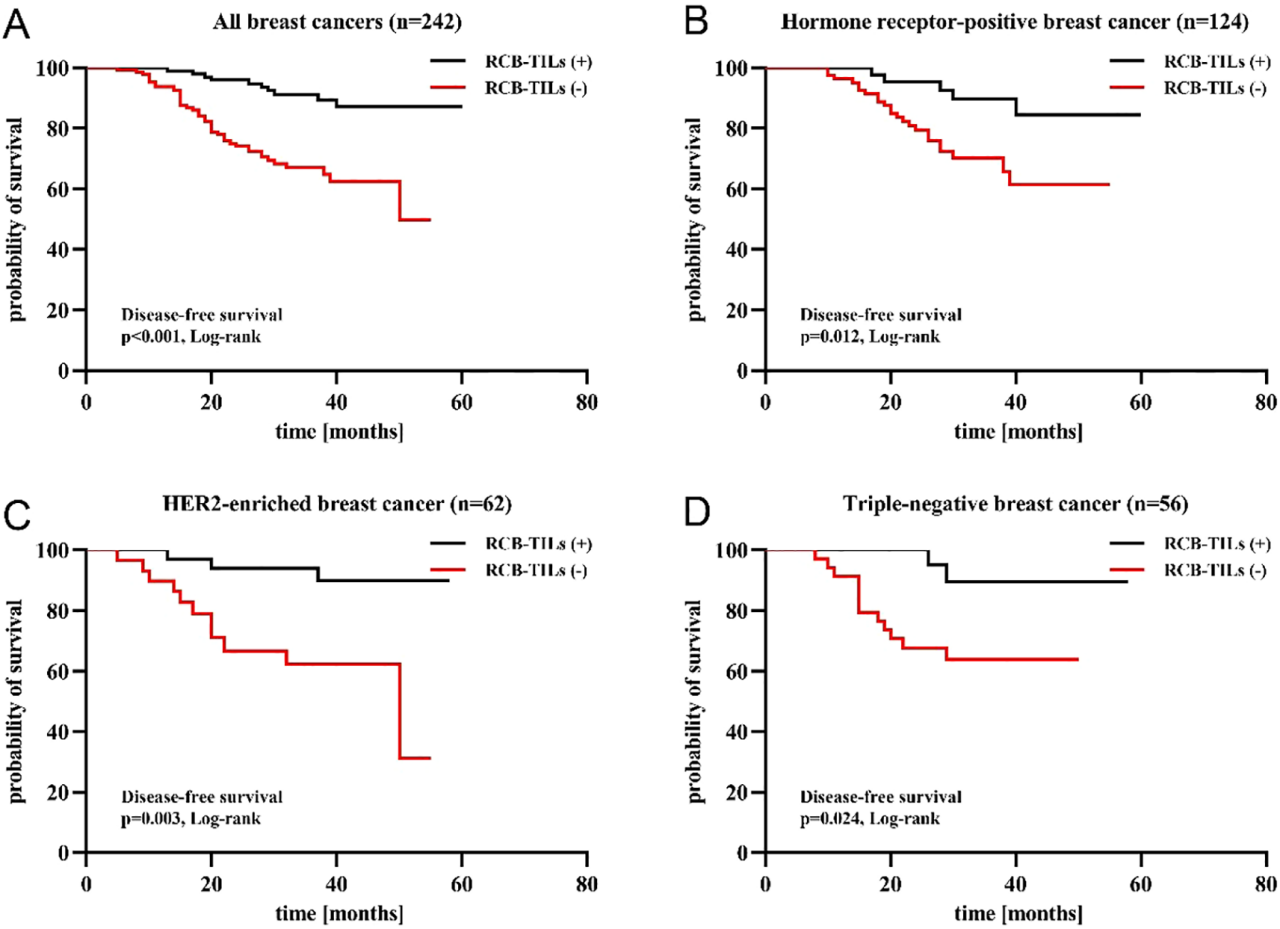
Kaplan–Meier analysis of disease-free survival in patients with breast cancer according to RCB-TILs **(A)** RCB-TILs in all breast cancer **(B)** RCB-TILs in HRBC **(C)** RCB-TILs in HER2BC **(D)** RCB-TILs in TNBC.

To comprehensively examine the prognostic significance of RCB-TILs in individuals with NAC BC, the Kaplan-Meier survival analysis was utilized to evaluate DFS. The findings revealed that the presence of RCB-TILs (+) was associated with a significant extension in DFS among all BC patients (P < 0.001), as well as within the subgroups of HRBC (P = 0.012), HER2BC (P = 0.003), and TNBC patients (P = 0.024) (**Figures 2A-D**).

Subsequently, both univariate and multivariate Cox analyses were conducted. The univariate Cox analysis indicated that TILs (+) were associated with an extension in DFS among all BC patients (HR = 0.447, P = 0.003) and HER2BC patients (HR = 0.288, P = 0.021), although no marked impact on survival was observed in HRBC patients (HR = 0.621, P = 0.215) or TNBC patients (HR = 0.324, P = 0.058). Conversely, RCB-TILs (+) were found to significantly contribute to extended DFS across all BC patients (HR = 0.239, P < 0.001), as well as within the HRBC (HR = 0.308, P = 0.018), HER2BC (HR = 0.181, P = 0.009), and TNBC subgroups (HR = 0.213, P = 0.043) (**Table 3**). The multivariate Cox analysis verified that RCB-TILs (+) functioned as an independent prognostic factor influencing recurrence following NAC in the entire cohort of BC patients (HR = 0.225, P < 0.001), and in the HRBC (HR = 0.213, P = 0.009), HER2BC (HR = 0.216, P = 0.045), and TNBC (HR = 0.220, P = 0.048) subgroups.

**Table 3:** Univariate and multivariate analysis of factors affecting DFS in NAC BC patients.

Characteristics		Univariable analysis			Multivariable analysis		
		Hazard ratio	95% CI	P value	Hazard ratio	95% CI	P value
All breast cancers (n=242)							
Age (years)	<60 vs ≥60	0.648	0.370-1.136	0.130			
Post-menopausal state	Yes vs No	0.647	0.378-1.106	0.112			
Tumor size	<2cm vs≥2cm	0.637	0.371-1.095	0.103			
Vascular invasion	Yes vs No	1.134	0.536-2.400	0.742			
Histological grade	I-II vs III	1.220	0.597-2.491	0.585			
Positive lymph node	Yes vs No	0.750	0.444-1.268	0.283			
Ki-67	<14% vs ≥14%	1.291	0.714-2.335	0.398			
Molecular subtype	HRBC vs non- HRBC	1.043	0.617-1.762	0.875			
Molecular subtype	HER2BC vs non-HER2BC	1.099	0.599-2.016	0.761			
Molecular subtype	TNBC vs non-TNBC	0.853	0.466-1.564	0.608			
Pathological response	PCR vs non-PCR	1.006	0.531-1.907	0.985	0.755	0.395-1.444	0.396
TILs	(-) vs (+)	0.447	0.262-0.764	0.003	1.023	0.545-1.921	0.943
RCB-TILs	(-) vs (+)	0.239	0.120-0.476	<0.001	0.225	0.099-0.508	<0.001
HRBC (n=124)							
Age (years)	<60 vs ≥60	0.489	0.198-1.208	0.121			
Post-menopausal state	Yes vs No	1.957	0.831-4.607	0.124			
Tumor size	<2cm vs≥2cm	0.553	0.261-1.170	0.121			
Vascular invasion	Yes vs No	3.084	0.730-13.026	0.126			
Histological grade	I-II vs III	1.194	0.283-5.047	0.809			
Positive lymph node	Yes vs No	0.650	0.304-1.389	0.266			
Ki-67	<14% vs ≥14%	0.977	0.430-2.220	0.956			
Pathological response	PCR vs non-PCR	0.800	0.324-1.976	0.628	0.525	0.205-1.346	0.180
TILs	(-) vs (+)	0.621	0.293-1.319	0.215	1.336	0.557-3.205	0.517
RCB-TILs	(-) vs (+)	0.308	0.117-0.815	0.018	0.213	0.067-0.682	0.009
HER2BC (n=62)							
Age (years)	<60 vs ≥60	0.714	0.246-2.067	0.534			
Post-menopausal state	Yes vs No	2.174	0.754-6.270	0.151			
Tumor size	<2cm vs≥2cm	0.978	0.269-3.557	0.973			
Vascular invasion	Yes vs No	2.232	0.612-8.147	0.224			
Histological grade	I-II vs III	1.212	0.268-5.484	0.803			
Positive lymph node	Yes vs No	0.841	0.277-2.552	0.759			
Ki-67	<14% vs ≥14%	3.855	0.860-17.285	0.078			
Pathological response	PCR vs non-PCR	2.714	0.602-12.232	0.194	1.908	0.396-9.184	0.421
TILs	(-) vs (+)	0.288	0.100-0.830	0.021	0.867	0.240-3.124	0.827
RCB-TILs	(-) vs (+)	0.181	0.050-0.650	0.009	0.216	0.048-0.968	0.045
TNBC (n=56)							
Age (years)	<60 vs ≥60	0.819	0.284-2.362	0.712			
Post-menopausal state	Yes vs No	0.819	0.274-2.445	0.720			
Tumor size	<2cm vs≥2cm	0.575	0.199-1.664	0.308			
Vascular invasion	Yes vs No	0.571	0.159-2.048	0.390			
Histological grade	I-II vs III	1.140	0.382-3.403	0.814			
Positive lymph node	Yes vs No	0.475	0.159-1.419	0.182			
Ki-67	<14% vs ≥14%	0.843	0.282-2.519	0.760			
Pathological response	PCR vs non-PCR	0.588	0.184-1.878	0.370	0.674	0.210-2.164	0.507
TILs	(-) vs (+)	0.324	0.101-1.037	0.058	0.766	0.173-3.398	0.726
RCB-TILs	(-) vs (+)	0.213	0.048-0.953	0.043	0.220	0.049-0.989	0.048

**Figure 3:**
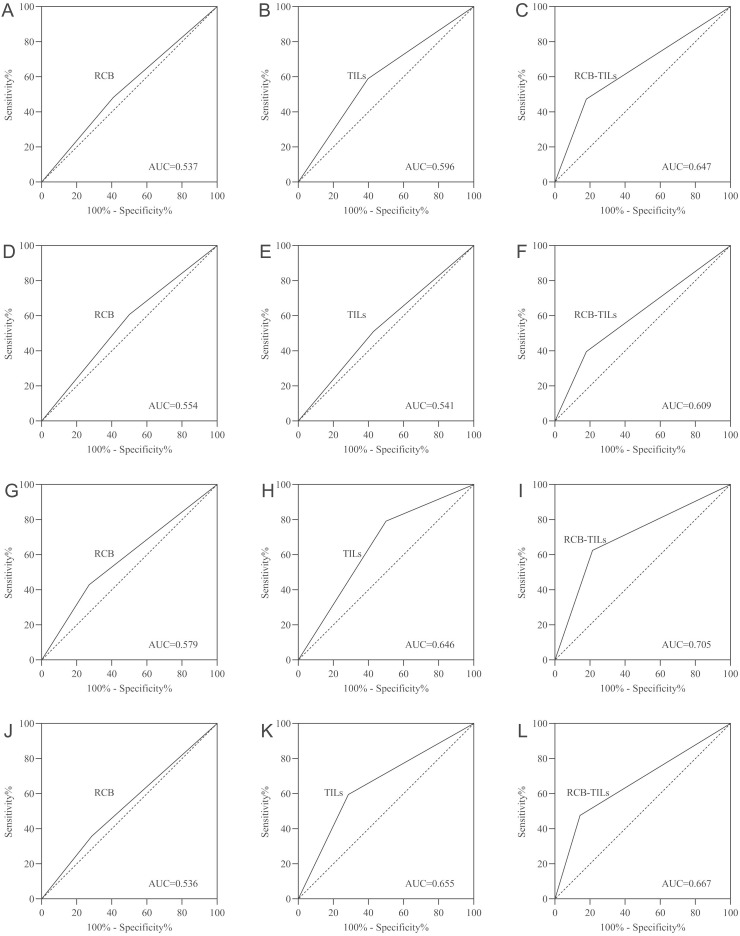
ROC curve analysis for all BC patients **(A-C)**, HRBC patients **(D-F)**, HER2BC patients **(G-I)**, and TNBC patients **(J-L)**.

Finally, a receiver operating characteristic (ROC) analysis was executed. The results indicated that RCB-TILs (area under the curve [AUC]: 0.647) surpassed both RCB (AUC: 0.537) and TILs (AUC: 0.596) in predicting outcomes for all BC patients (**Figures 3A-C**). Additional analyses by subtype showed consistent results in HRBC patients (AUC: RCB-TILs = 0.609, RCB = 0.554, TILs = 0.541) (**Figures 3D-F**), HER2BC patients (AUC: RCB-TILs = 0.705, RCB = 0.579, TILs = 0.646) (**Figures 3G-I**), and TNBC patients (AUC: RCB-TILs = 0.667, RCB = 0.536, TILs = 0.655) (**Figures 3J-L**).

## 4 Discussion

The utilization of NAC in BC treatment has become increasingly prevalent. By decreasing the staging of primary breast tumors and axillary lymph node metastases, NAC can effectively control disease progression, thereby enhancing patient survival rates and prognosis (14). Consequently, an accurate and effective prognostic indicator is vital for the diagnostic and therapeutic evaluation of BC patients undergoing NAC. PCR, characterized by the lack of invasive and *in situ* residual disease in both breast and lymph nodes, assists in identifying patients with favorable and unfavorable outcomes (15). Although PCR is correlated with a positive prognosis in HER2BC and TNBC subtypes, it is not suitable for prognostic assessment in HRBC subtype patients (15). Previous studies have demonstrated (16) that TILs can serve as an evaluation indicator for predicting the efficacy of TCHP regimen treatment in HER2BC patients. Additionally, the work of Hou Z et al. (8) confirmed that elevated levels of TIL infiltration in tumor tissue prolonged DFS and overall survival in non-small cell lung cancer patients receiving NAC treatment. Although TIL evaluation has shown good efficacy in predicting NAC treatment response for TNBC and HER2BC patients, satisfactory results are often challenging to obtain for predicting treatment response in the most common HRBC subtype (17). Furthermore, a multicenter analysis involving 5,161 patients indicated that post-NAC RCB assessment could be employed to predict survival in HRBC patients (6). Sano Y et al. (17) discovered that combining TIL assessment with RCB scoring could effectively enhance the predictive performance of the RCB assessment system. Therefore, this study integrated RCB with TILs to evaluate the RCB-TILs status of NAC BC patients diagnosed and treated at three medical centers, aiming to assess its effectiveness as a survival predictor for these patients.

TILs exert specific cytotoxic effects on tumor cells and are considered markers of highly immunogenic subtypes (18). In this study, the RCB-TILs (+) BC group exhibited reduced rates of vascular invasion and lymph node metastasis in comparison to the RCB-TILs (-) group, suggesting that elevated levels of TILs might exert a substantial influence on suppressing tumor cell proliferation and metastasis. Furthermore, a higher PCR rate was noted in the RCB-TILs (+) group, suggesting that patients with RCB-TILs (+) status were more likely to achieve PCR compared to those with RCB-TILs (-), potentially implying improved survival outcomes for RCB-TILs (+) patients. Some studies have proposed that RCB-TILs serve as a critical predictor of recurrence for all invasive BCs following NAC and could function as an effective indicator of NAC efficacy. It has also been observed that the TNBC subtype contains a higher proportion of RCB-TILs (+) cases compared to other subtypes (17). However, in this investigation, a higher percentage of RCB-TILs (+) was identified in the HER2BC subtype, which might be attributable to the relatively larger number of HER2BC subtype patients included or variations in the genetic backgrounds of the study subjects. This finding warrants further verification in future research. Multivariate Cox analysis was employed to evaluate survival across all BC subtypes, revealing that RCB-TILs (+) constitute a favorable factor for extended DFS in BC patients post-NAC. Moreover, ROC analysis demonstrated that RCB-TILs are a more sensitive predictor of survival compared to using RCB or TILs independently. Consequently, RCB-TILs hold promise as a predictor of post-NAC survival for patients with various BC subtypes. When contemplating additional treatment following NAC, RCB-TILs assessment may aid in formulating more suitable treatment strategies. Despite expressing ER or PR, some BC patients do not respond to endocrine therapy, while others develop resistance during treatment (19). In this study, all HRBC subtype patients who underwent NAC also received subsequent endocrine therapy. RCB-TILs (+) patients exhibited lower recurrence rates, suggesting that RCB-TILs could potentially serve as an alternative indicator for predicting endocrine therapy response in HRBC subtype patients. In light of this, some researchers have advocated a new treatment strategy where HRBC subtype patients with RCB-TILs (-) status could be considered for additional chemotherapy alongside standard endocrine therapy (17). Masuda N et al. (20) reported on a clinical trial applying capecitabine to HER2-negative BC patients after NAC and surgery. It is anticipated that future similar studies will also examine the correlation between RCB-TILs and prognosis in BC patients post-NAC. Although the effectiveness of RCB-TILs in predicting survival for BC patients after NAC has been evaluated through a three-center study, further research is necessary to ascertain whether RCB-TILs are equally applicable in other ethnic groups.

## 5 Conclusion

This study illustrates that RCB-TILs are linked to survival outcomes in BC patients undergoing NAC, potentially serving as a more sensitive predictor of recurrence than using RCB or TILs independently. Furthermore, RCB-TILs exhibit promise as a potential biomarker for identifying DFS in BC patients treated with NAC, offering valuable guidance for subsequent clinical diagnosis, treatment, and evaluation.

## Data Availability

The original contributions presented in the study are included in the article/Supplementary Material. Further inquiries can be directed to the corresponding author.
